# The polymorphism of EGFL6 D535N is not associated with obesity in Chinese children

**DOI:** 10.1186/1687-9856-2013-S1-P106

**Published:** 2013-10-03

**Authors:** Chun Lin Wang, Yi Dong Wu, Li Liang, Chao Chun Zou, Jun Fen Fu

**Affiliations:** 1 Department of Endocrinology, the Children’s Hospital of Zhejiang University School of Medicine, Hangzhou, China; 2 The Key Laboratory of Reproductive Genetics (Zhejiang University), Ministry of Education Hangzhou, China

**Keywords:** Epidermal growth factor-like domain multiple-6 (EGFL6), Polymorphism, Mutation, Obesity, Children, Chinese

## Objective

To investigate the association between EGFL6 D535N polymorphism and obesity in Chinese children.

## Design

Case-control study.

## Subjects

A total of 384 obese and 205 healthy children were enrolled as obese and control groups.

## Measurements

The tagged SNP in EGFL6 (rs16979033) was measured by automated platform MassArray. Anthropometric estimates (BMI, BMI Z score, waist circumference, waist-height-ratio) and biochemistry marker measurements (fasting glucose, fasting insulin, total cholesterol, triglyceride, HDL-C, non-HDL, HOMA-IR ) were performed.

## Results

The frequencies of the N allele were 13.9% in 589 children. Among the obese group, 329 were homozygous for the 535D allele, 22 girls were heterozygous and 33 were homozygous for the 535N allele. In the control group, 173 were homozygous for the 535D allele, 15 girls were heterozygous and 17 were homozygous for the 535N allele. The frequencies of the N allele were 13.5% in obese group and 14.5% in control group, having no significant difference (χ^2^=0.119, p=0.730). The EGFL6 D535N polymorphism was not associated with obesity in either the dominant or recessive model test. The anthropometric estimates and biochemistry markers in patients with EGFL6 353D were not significantly different than those in patients with EGFL6 353N

## Conclusion

The polymorphism of EGFL6 D535N was very common in Chinese children. EGFL6 D535N polymorphism was not associated with obesity in Chinese children and it may not be a cause of childhood obesity.

